# A Versatile Protocol for Efficient Transformation and Regeneration in Mega *Indica* Rice Cultivar MTU1010: Optimization through Hormonal Variables

**DOI:** 10.3390/mps6060113

**Published:** 2023-11-23

**Authors:** Pragya Yadav, V. V. Santosh Kumar, Jyoti Priya, Shashank Kumar Yadav, Shivani Nagar, Meenu Singh, Viswanathan Chinnusamy

**Affiliations:** 1 Division of Plant Physiology, ICAR-Indian Agricultural Research Institute (IARI), New Delhi 110012, India; pragya_physio@iari.res.in (P.Y.); santoshk_physio@iari.res.in (V.V.S.K.); jyoti.priya@icar.gov.in (J.P.); shashank_physio@iari.res.in (S.K.Y.); shivani.nagar@icar.gov.in (S.N.); 2 Department of Biotechnology, College of Engineering and Technology, IILM University, Greater Noida 201310, India; meenu.singh@iilm.edu

**Keywords:** in vitro culture, callus induction efficiency, transformation efficiency, regeneration efficiency, BAP, kinetin, NAA

## Abstract

Rice is one of the apex food crops in terms of meeting the daily calorific and dietary requirement of the majority of the world population. However, rice productivity is severely limited by various biotic and abiotic attributes, causing a severe threat to global food security. In the use of functional genomics and genome editing for the generation of trait-enhanced genotypes, it is necessary to have an efficient genetic transformation and regeneration protocol. The recalcitrant nature and paucity of efficient and versatile genetic transformation and regeneration protocols for *indica* cultivars remains a constraint. In the present study, we have optimized a tissue culture method for MTU1010, a mega *indica* rice variety. We conducted a combinatorial analysis of different plant growth regulators on embryogenic callus induction efficiency, and it was observed that MSB5 medium supplemented with 2.5 mg/L 2-4D and 0.25 mg/L 6-BAP results in maximum embryogenic callus induction, i.e., 92%. The regeneration efficiency of a transformed callus can be enhanced by up to 50% with the supplementation of 1 mg/L kinetin alongside 2.5 mg/L BAP and 0.5 mg/L NAA in the shooting medium. Furthermore, our results unveiled that the pre-activation of *Agrobacterium* culture for 30 min with 150 µM acetosyringone significantly increased the transformation efficiency of calli. Additionally, descaling the salt concentration to half strength in resuspension and co-cultivation increased the efficiency of transformation up to 33%. Thus, the protocol developed in this study will be instrumental for the genome editing and genetic engineering of *indica* rice cultivars for functional genomics studies and crop improvement.

## 1. Introduction

Rice is the principal food crop that provides food for more than 2.5 billion people and is the second-most cultivated crop of the world [1,2]. Globally, rice is cultivated on nearly 162 million hectares with an annual production of about 756 million tonnes (FAO, 2022). The burgeoning population demands a sustainable and improved system of rice cultivation with more efficient cultivars to ensure the production of 852 million metric tonnes by 2035 [3]. The significance of rice as a staple food crop is evident from the fact that >50% of the world and 80% of the Asian population depend on rice for their daily dietary requirement [4,5,6,7]. The productivity and quality of rice is governed by several factors, including genetic potential and environmental constraints [8,9,10]. Different traditional and modern approaches have been applied to improve the productivity of rice, namely mutation breeding [11], hybrid breeding [12,13], marker-assisted breeding [14], genetic engineering, and genome editing [15]. Elite rice genotypes which can tolerate extreme environmental conditions can be propagated through combining conventional and modern genetic manipulation approaches, using in vitro tissue culture methods [16]. This leads to the acceleration of genetic gains, which significantly increases the productivity and quality. Under these challenges, rice productivity must be doubled for sustainable food security by 2050. The current rate of genetic gain in yield is 1%, which needs to be increased to 2.4% by 2050. Therefore, finding new breeding technologies such as genome editing for crop improvement is imperative for sustainable agriculture. Among all species of rice, *indica* rice occupies a paramount position owing to prominent demands in the subtropics and tropics. Rice genotypes, primarily those belonging to the *indica* subspecies, are recalcitrant to genetic transformation and regeneration techniques, thereby making various functional genomics and trait enrichment endeavors formidable tasks. Thus, *indica* rice attracts the attention of researchers from across the globe for the optimization of an efficient genetic transformation regeneration system.

Cotton Dora Sannalu (MTU 1010) is an important semi dwarf rice cultivar with a long slender grain type and is tolerant to BPH and blast disease, respectively. It is predominantly cultivated in tropical and subtropical agroclimatic zones and is widely used as a parent in several trait improvement breeding programs. In India, currently more than 375 varieties are in seed chain, of which MTU1010, one of the mega rice varieties of India, occupies approximately 6% of the total rice cultivating area in India, contributing substantially to the national food pool as well as generating Rs from 1200 to 1500 crores in income every year [17]. Despite several transformation and regeneration protocols having been formulated and standardized in *japonica* varieties, limited work has been undertaken to enhance the efficiency of the transformation and regeneration of *indica* rice genotypes. Therefore, efficient *Agrobacterium*-mediated genetic transformation protocols for *indica* rice genotypes, with a high frequency of regeneration and reproducibility, need to be developed.

With the recent advancement of several functional genomics approaches generating largely annotated and high-quality genomic data resources for genetic manipulation, the characterization and functional validation of several genes are necessary for trait engineering. This necessitates a rapid, reproducible, and efficient genetic transformation method. *Agrobacterium*-mediated gene transfer is among the most commonly used transformation methods and can be reproduced easily and has easy handling and operation [18,19]. The stable and low copy number integration of T-DNA in plant chromosomes gives *Agrobacterium*-mediated gene transfer an additional advantage over other genetic transformation systems. However, it requires efficient regeneration from the transformed callus which is difficult in most of the *indica* varieties. The standardization of existing *Agrobacterium*-mediated genetic transformation protocols like efficient T-DNA delivery, several factors like concentration of acetosyringone, pH, salt concentration, selection of plant growth regulators, proportion of gelling agent, carbon source, and photoperiodic incubation, and optimum growth conditions of *Agrobacterium* culture in mega rice cultivar are the need of the hour.

Many studies have been conducted involving different types of media, namely MS, N6, L3, Dl, B5, AA, along with altered concentrations of macro and micro salts present therein [20,21]. Furthermore, different concentrations of gelling agents have been used to impart desiccation to the calli in regeneration media [22,23,24]. However, these protocols have a consequential bottleneck in terms of transformation and regeneration efficiency. Present study aims to establish a rapid, reproducible, efficient, and optimized protocol for a mega variety of *indica* rice, MTU1010, for better callus induction, higher transformation efficiency, and regeneration of the transformed callus through altering various factors in tissue culture methods.

## 2. Materials and Methods

### 2.1. Plant Material

Mature seeds of MTU1010 produced at the experimental farm of the ICAR-Indian Agricultural Research Institute, New Delhi were used as explant in present study and embryogenic calli were used as the target material for optimization of transformation and regeneration protocol.

### 2.2. Procedure

#### 2.2.1. Surface Sterilization of Mature Rice Seeds

Healthy and mature seeds of MTU1010 were selected by physical appearance and de-husked manually. Seeds were prewashed 2–3 times with autoclaved distilled water and then surface-sterilized with 70% ethanol (*v*/*v*) for 90 s, followed by a rinse with autoclaved distilled water. These seeds were then sterilized with 2% (*v*/*v*) sodium hypochlorite and 0.05% nonionic detergent (Tween 20/Triton x-100) with gentle shaking for 20 min. Seeds were then washed 8–10 times with autoclaved distilled water, and air-dried on autoclaved Whatman paper for about an hour. These seeds were cultured on MS [25] media with an array of different combinations of growth regulators like cytokinins and auxins.

#### 2.2.2. Optimization of Efficient Callus Induction and Regeneration in Rice cv. MTU1010

To test the effect of different plant growth regulators (PGR) on callus induction frequency of MTU1010, surface-sterilized seeds were inoculated on callus induction medium (CIM) supplemented with 1.5, 2.5, and 3.5 mg/L of 2,4-D alone and in combinations of 1.5, 2.5, and 3.5 mg/L of 2,4-D and 0.15 and 0.25 mg/L of 6-BAP. Phytagel™ (Sigma) 0.4% was used as gelling agent and pH adjusted to 5.8 prior autoclaving. Growth regulator(s) and other supplements were added post-autoclaving to the media at slightly higher ambient temperature. Upon congealing, 18–20 seeds were inoculated per plate, and incubated at 27 ± 1 °C under dark conditions. After 13–14 days, newly grown calli were sub-cultured in fresh media plates and grown for another 7 days. Data for callus induction frequency were recorded after 3 weeks to select the best combination of the media suited for MTU1010 variety. Subsequently, the best-suited combination of calli was used to test the regeneration potential in MTU1010. In this study, two different combinations of regeneration media were used, i.e., 2.5 mg/L BAP, 1 mg/L kinetin, and 0.5 mg/L NAA and 2.5 mg/L BAP and 0.5 mg/L NAA. Callus growth and morphology was observed under stereo microscope (Carl-Zeiss, Baden-Württemberg, Germany).

#### 2.2.3. Gene Constructs and Agrobacterium Strain Used for Rice Transformation

**Figure 1:**
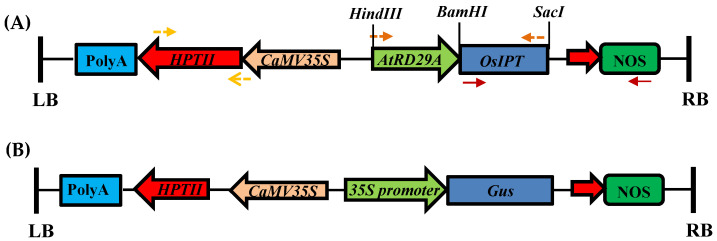
Schematic representation of the gene construct. *OsIPT* cloned in pCAMBIA1300 plant transformation vector (**A**) and linear map of binary vector pCAMBIA1301 T-DNA region (**B**).

The transgene used in present study is rice Isopentenyl transferase gene (*OsIPT9*), which was cloned in pCAMBIA1300 plant transformation vector under transcriptional control of stress inducible promoter. pCAMBIA1300-*P_AtRD29A_::OsIPT9*, having *nptII* (neomycin phosphotransferase) and *hptII* (hygromycin phosphotransferase) gene as bacterial and plant selection marker, respectively, were used. Likewise, additional gene construct, pC1301, harbouring *uidA* encoding GUS as the reporter gene was used for transformation efficiency assaying study (Figure 1). Both the gene constructs were transformed in *Agrobacterium tumefaciens* strain EHA105.

#### 2.2.4. Preparation of Primary and Secondary Agrobacterium Culture

Primary culture was prepared by inoculating single colony of both gene constructs from a freshly streaked plate in 5 mL of autoclaved yeast extract mannitol (YEM) medium comprising 12.5 mg/L rifampicin and 50 mg/L kanamycin. The culture was incubated at 28 °C for 24–36 h at 180 rpm in an incubator shaker (Kuhner, Basel, Switzerland). Further, for secondary culture, 0.1–0.5%, depending on the growth, was added to 100 mL of YEM media supplemented with antibiotics.

#### 2.2.5. Transformation and Co-Cultivation

Once the Optical Density (O.D.) of secondary culture reached ~0.6–0.8, *Agrobacterium* culture (EHA105) was pelleted in Oak-Ridge tubes by centrifugation at 4000 rpm at 25 °C for 20 min. The pellet was resuspended in 20–30 mL of bacterial resuspension medium (RSM with 1.5% sucrose, pH-5.4) containing 150 µM acetosyringone and kept for pre-activation at 27 ± 1 °C for at least 25–30 min in the dark in an incubator prior to calli infection. Generally, batch of 100 embryogenic calli was used for one transformation, and was then immersed in bacterial resuspension medium with gentle shaking at 60 rpm in incubator shaker for 20 min. The Agro-infected calli were air dried on sterile Whatman paper for 8–10 min only. Agro-infected calli were then transferred onto co-cultivation medium containing 150 µM acetosyringone and incubated in dark at 27 ± 1 °C for 48 h. In this study, three different combinations of resuspension and co-cultivation media were used, i.e., Full MS, ½ MS, and ¼ MS, and the best suited combination of MS salt was used for infection media in MTU1010.

#### 2.2.6. Washing of Calli and Resting Medium

After 48 h of incubation, slight growth of *Agrobacterium* appeared around the calli, which were gently rinsed 6–8 times with autoclaved distilled water on visual observation until turbidity disappeared. Further, the calli were rinsed with 25–30 mL of autoclaved water containing 300 mg/L cefotaxime and 200 mg/L Timentin three times at 28 °C for 20 min at 60 rpm and dried on Whatman paper for 1.5–2 h. The calli were then intermediately transferred onto a resting medium (CIM containing 300 mg/L Cefotaxime and 200 mg/L Timentin) and incubated in dark at 27 ± 1 °C for around 5–7 days.

#### 2.2.7. Selection of Transformed Calli

After 7 days of resting, infected calli were transferred to the first selection medium, SM-1 (14–15 calli/plate) containing 300 mg/L Cefotaxime, 200 mg/L Timentin, and 50 mg/L Hygromycin (Hi-media), and incubated in dark at 27 ± 1 °C for 15 days. After 15 days of 1st selection, infected calli were transferred to freshly prepared selection medium-1 and incubated for another 15 days in dark. Post two rounds of selection, brown or black calli were removed and only the embryogenic white microcalli were transferred onto fresh SM-2 containing 250 mg/L Cefotaxime and 50 mg/L hygromycin, and allowed to proliferate at 27 ± 1 °C for 7–10 days in dark for final selection.

#### 2.2.8. Regeneration of Transformed Calli

After residing 40 days in selection medium, resistant proliferated white calli were transferred onto regeneration medium comprising MS salts, 30 g/L maltose, 2.5 mg/L BAP, 1 mg/L Kinetin, 0.5 mg/L naphthalene acetic acid (NAA), 250 mg/L cefotaxime, and 35 mg/L hygromycin with 10 g/L agarose. Different combinations and concentrations of growth regulators and media components which were optimized have been mentioned in Table 1 and Table S1. These microcalli were incubated at 27 ± 1 °C for 7 days in dark for organogenesis. After that transfer, the white calli were proliferated in fresh regeneration medium with 8 g/L agarose for 12–15 days in light (16 h/8 h day/night) photoperiod.

#### 2.2.9. Rooting Medium and Root Hardening

**Figure 2:**
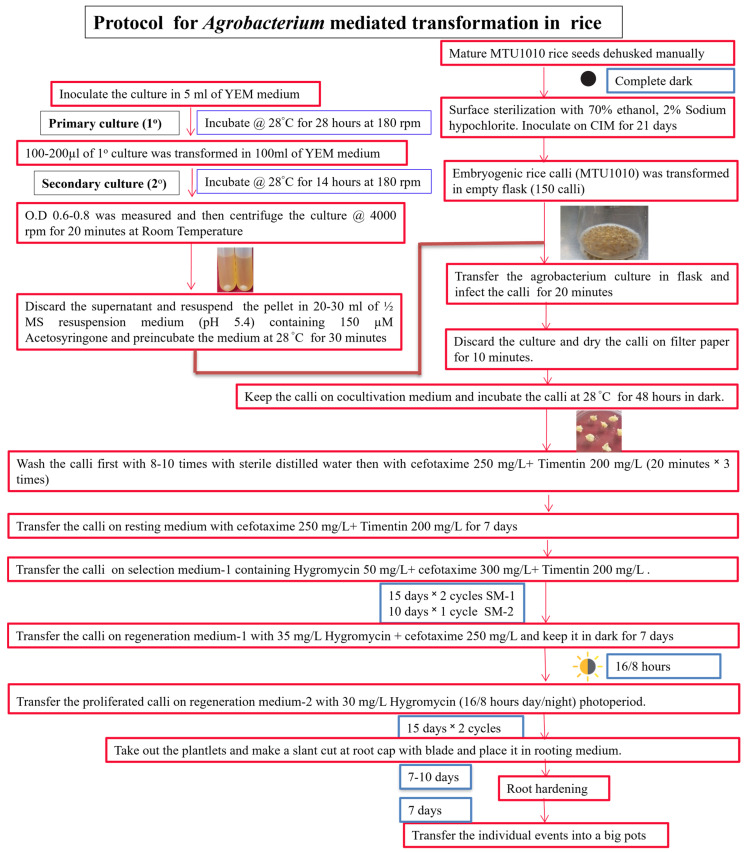
Schematic flowchart of steps involved in *Agrobacterium*-mediated transformation in MTU1010 for regeneration of transgenic plants. The ‘red boxes’ represents the steps or procedure while the ‘blue box’ represents the growth condition/durations.

Regenerated shoots were transferred to rooting media (½ MS salts, 20 g/L Sucrose, 10 g/L Glucose, pH-5.8, and 2.5 g/L Phytagel) and kept in light/dark (16/8 h) for 7–10 days. Prior to transferring the regenerated shoot, a slant (fine cut) was made in root cap region using scalpel blade. Upon induction of roots, the putative transformed plants were transferred to half-strength Yoshida solution [26] to enhance the root growth for one week. Later, the plants were transferred to soil rite^®^ mixture in pots for 7–10 days and further transplanted to soil and maintained in transgenic glass house (IARI, New Delhi, India). The flowchart summarizing all above essential steps has been mentioned in (Figure 2).

#### 2.2.10. Molecular Confirmation of Putative Transgenic Plants

Total genomic DNA was isolated from various individualistic putative transgenic plants using cetyltrimethylammonium bromide (CTAB) method [27] and 50–100 ng of DNA was used as a template for PCR amplification using *hptII* gene specific primers (forward 5′GAGAGCCTGACCTATTGCATCTC3′ and reverse 5′GTCAACCAAGCTTTCTGATAGAGTTG3′. Plasmid DNA (pCAMBIA1300-*P_AtRD29A_::OsIPT9*), which were used as positive control, while DNA of non-transformed wild type MTU1010 plants were used as negative control. The PCR product was run on 1.5% TAE agarose gel and visualized under gel documentation system.

#### 2.2.11. Southern Blotting

Genomic DNA was isolated from the (T_0_) *OsIPT9* transgenic plants following CTAB method [27,28]. For Southern blotting, 15 µg of DNA was digested with *Sac*I and the digested samples were resolved on 0.8% agarose gels electrophoresis with 1X TAE buffer at 40 volts for 5–6 h. DNA was transferred from the gel into to negatively charged nylon Hybond Nylon+ membrane (Hybond-N+, Roche) using capillary method. The blots were hybridized with probe synthesized using PCR DIG Probe Synthesis Kit (Roche Diagnostics, Mannheim, Germany) using *hptII* specific primers. Hybridization was done at 42 °C overnight and washed the next day. Anti-DIG Fab Fragment-AP conjugate (Roche Diagnostics, Mannheim, Germany) was used to detect the DIG labeled probe. The blots were detected using CDP-Star Detection Reagent (Roche Diagnostics, Mannheim, Germany) following manufacturers protocols.

#### 2.2.12. β-Glucuronidase Staining (GUS) Assay

The pC1301-transformed calli of different treatments and control calli were histochemically assayed for GUS staining using the method described by [29]. After co-cultivation, the calli were washed and then immersed in GUS solution and incubated overnight at 37 °C. For qualitative assay, the area of GUS expression and intensity of the blue color at each calli observed.

#### 2.2.13. Statistical Analysis

All experiments were repeated in at least three biological replicates for each treatment. One way ANOVA was carried out for each experiment and data were considered statistically significant at a *p* < 0.001. Graphpad *t*-test analyser (https://www.graphpad.com/quickcalcs/ttest1.cfm, accessed on 18 June 2023) has been used for statistical analysis. Asterisks (*) indicate statistically significant differences (*p* < 0.001).

## 3. Results and Discussion

While establishing a successful transformation system for a specific plant variety, the foremost requirement is to emplace a stable and efficient plant regeneration protocol. The competence of calli for transformation is predominantly determined by the conditions existing during the co-cultivation of embryogenic calli. Our work is primarily focused on improving the diverse aspects of the infection stage, chiefly by exploring factors like the effects of preincubation duration, and the different salt concentrations in co-cultivation and resuspension media. Additionally, we have also investigated the role of varied hormonal combinations and concentrations that were optimized in CIM for enhanced embryogenicity of the calli as well as in shoot regeneration efficiency. Thus, a protocol for efficient regeneration and transformation for MTU1010 rice variety has been formulated in the present study (Figure 2).

### 3.1. Optimization of In Vitro Embryogenic Callus Induction in Rice cv. MTU1010

**Figure 3:**
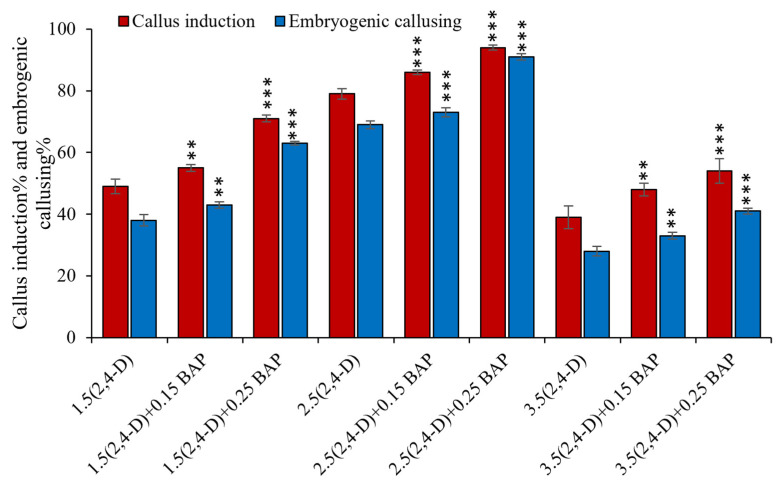
Optimization of PGR concentrations for callus induction in MTU1010. Data shown are ± SE (standard error) of three individual experiments. Asterisks (**, ***) indicate statistically significant differences (*p* < 0.001). Media augmented only with 2,4-D is used as a calibrator for individual concentration used.

**Figure 4:**
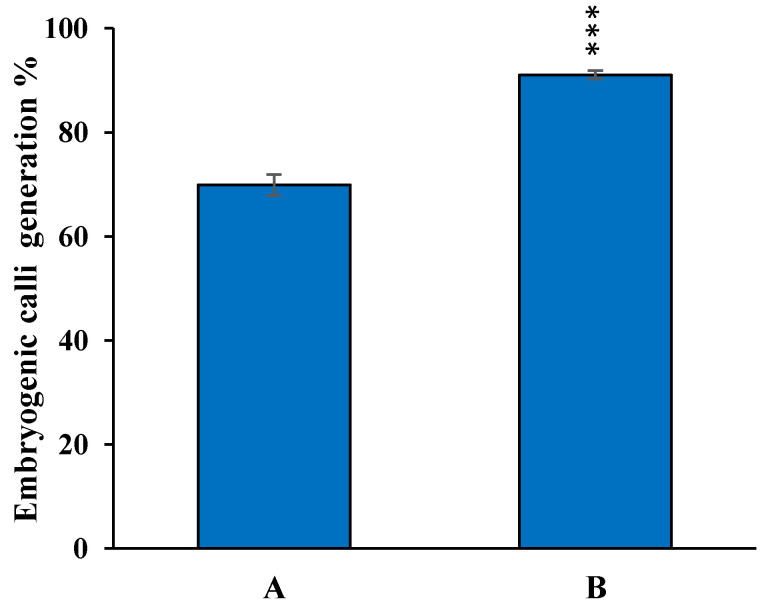
Effect of BAP on embryogenic callus induction frequency on callus induction media. Data shown are ± SE (standard error) three individual experiments. A, CIM + 2.5 mg/L 2,4-D; B, CIM+ 2.5 mg/L 2,4-D + 0.25 mg/L BAP. Asterisks (***) indicate statistically significant differences (*p* < 0.001).

It was observed that 2,4-D at 2.5 mg/L concentration was required for an efficient callus induction rate (over 75%), wherein the presence or absence of BAP supplementation had an insignificant or minimal effect on the callusing efficiency (Figure 3). However, the embryogenicity of the callus was markedly enhanced when using CIM supplemented with 0.25 mg/L BAP in addition to 2.5 mg/L of 2,4-D. Out of three different BAP concentrations that were studied, 0.25 mg/L BAP was found to be the most yielding towards the generation of embryogenic calli, with around ~92%. (Figure 4; Supplementary Table S2).

**Figure 5:**
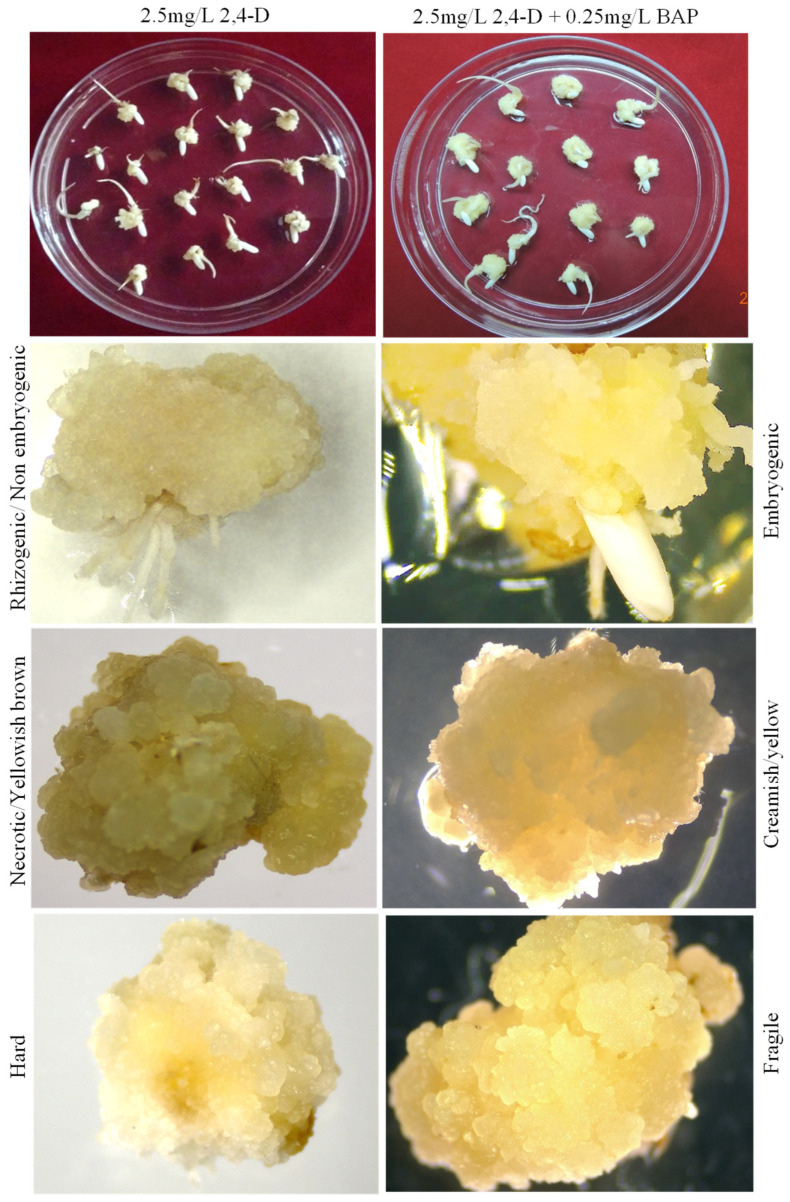
Morphological characteristics of callus in two different hormone concentrations, revealing the effect of BAP on calli quality on CIM media. Left panel (CIM with only 2.5 mg/L 2,4-D) shows non-embryogenic while the right panel (CIM with 2.5 mg/L 2,4-D and 0.25 mg/L BAP) shows embryogenic calli of rice cultivar MTU1010.

Additionally, the morphologies of embryogenic calli were discerned using a Stereo microscope (Carl-Zeiss, Baden-Württemberg, Germany), revealing calli that were more whitish, fragile, and embryogenic in appearance in media having both 2.5 mg/L 2,4-D and 0.25 mg/L BAP, while the presence of hard, yellowish, necrotic, hairy, and non-embryogenic calli were witnessed in media containing only 2.5 mg/L 2,4-D (Figure 5). Consequently, embryogenic calli were scrutinized from non-embryogenic calli for an efficient regeneration and genetic transformation efficiency of rice. The results suggested that CIM supplemented with 2.5 mg/L 2,4-D and 0.25 mg/L 6-BAP was the best-suited media for the successful genetic transformation of MTU1010 rice. Several carbon sources were evaluated in different amalgamations and maltose was decisively preferred for MTU1010, primarily during the early stages involving callus induction and shoot proliferation. The maltose-supplemented media exhibited a higher differentiation degree and ultimately plant regeneration owing to the lower osmotic potential of maltose compared to other sugars like sucrose and glucose [22,30,31,32,33]. Further, maltose has been reported to promote somatic embryogenesis and enhance shoot regeneration while effectively inhibiting phenolic secretion [23].

### 3.2. Downscaling Salt Concentration during Infection and Co-Cultivation Can Enhance the T-DNA Delivery and High Transient Expression Efficiency in Rice cv. MTU1010

For efficient T-DNA delivery, the salt concentration used in the co-cultivation and resuspension media plays a pivotal role in the successful genetic transformation of rice. In crops such as maize, canola, and wheat, the use of a low salt concentration in resuspension and co-cultivation medium during infection has been reported to enhance the T-DNA delivery, which in turn increases the transformation efficiency [34,35,36].

To optimize the T-DNA delivery of *Agrobacterium*, the two types of resuspension media (RSM1 and RSM2) and co-cultivation media (CCM1 and CCM2) were tested to evaluate the regeneration and transformation efficiency of the MTU1010 genotype. Also, several days-old calli were used for the estimation of co-cultivation and transformation efficiency, amongst which 21-days-old calli were found to be the most apt for downstream processes. Previously, different concentrations of acetosyringone in RSM were studied and 150 µM was selected with highest transformation frequency. Further, it might also act as a bacteriostatic at higher concentrations [37].

**Figure 6:**
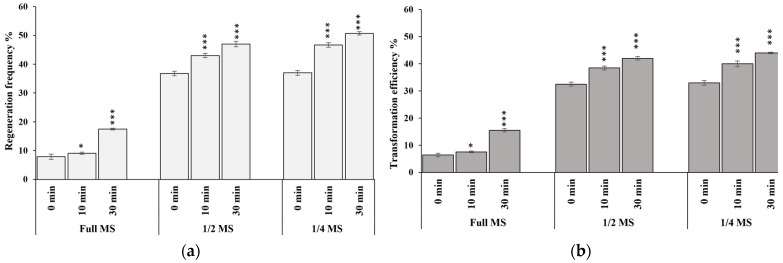
(**a**,**b**) Regeneration and transformation efficiency of indica rice cultivar MTU1010 in different resuspension mediums at different pre-incubation times during *Agrobacterium*-mediated rice transformation. Data shown are mean of three individual experiments. Asterisks (*, ***) indicate statistically significant differences (*p* < 0.001).

It was observed that, for the genetic transformation of cv. MTU1010 rice with resuspension medium-1 (RSM-1) and co-cultivation medium-1 (CCM-1), the maximum regeneration efficiency of 17% and maximum transformation efficiency of 15% were achieved in 30 min of pre-activation. In case of RSM-2 and CCM-2 media, the maximum regeneration efficiencies of 46% and 50%, and transformation efficiencies of 42% and 44% were achieved with 30 min of pre-activation (Figure 6; Supplementary Table S3).

**Figure 7:**
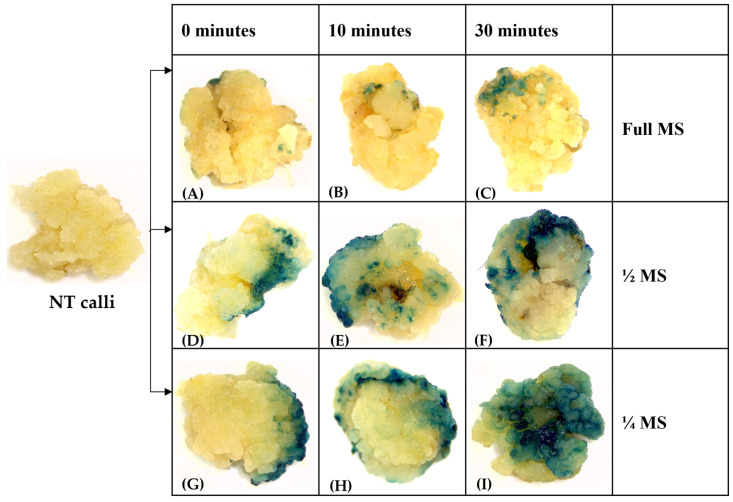
GUS gene expression in calli transformed with pC1301 construct in different MS salt strengths. [(**A**–**C**): Calli co-cultivate in full strength MS; (**D**–**F**): co-cultivate in half strength (_1/2_) MS salt; (**G**–**I**): co-cultivate in one-fourth strength (_1/4_) MS salt] at different pre-incubation times. Transformed calli (25 calli) were stained after two days of co-cultivation. Pictures show representative calli from two independent transformations.

The transformation efficiency was assessed for the transformed calli using GUS staining assay. The results revealed that decreasing MS salt concentration from full strength to half and one-fourth strength increased GUS induction. Further, the 30–40 min pre-activation of *Agrobacterium* culture with acetosyringone improved the transformation efficiency. The GUS staining showed a lower number of GUS-stained cells in full MS (RSM-1), while the complete cell cluster expressed the GUS gene in the half and one-fourth strengths of MS media (RSM-2) (Figure 7). Thus, an optimum salt concentration in the co-cultivation medium plays a crucial role in the successful co-cultivation of *Agrobacterium* in rice tissue culture. An optimal salt concentration ensures osmotic potential, which is conducive to both the bacteria and the plant cells, promoting their interaction and enhancing the transfer of T-DNA (transfer DNA) into the plant cells. High salt concentrations can induce osmotic stress in the bacteria, leading to cell dehydration and reduced survival. The salt concentration also influences the attachment efficiency to the plant surface of *Agrobacterium*.

### 3.3. Effect of Pre-Incubation on Transformation Efficiency

The activation of the *Vir* operon of *Agrobacterium* is a prerequisite for the systematic and efficient transfer of T-DNA into plant chromosomes. Here, we have optimized the preincubation time of *Agrobacterium* culture with acetosyringone to improve the infection rate [38]. The *Agrobacterium Vir* genes can be induced using 150 µM Acetosyringone at a lower pH of 5.4 in preculture medium (*Agrobacterium* culture in RSM) before the inoculation/co-cultivation of bacteria on calli. The present study showed that the preincubation of *Agrobacterium* culture combined with half and one-fourth salt concentrations both in co-cultivation and resuspension medium resulted in the maximum transformations of about 42% and 44%, respectively, in 30 min of pre-activation (Figure 6; Supplementary Table S3). Preincubations of *Agrobacterium* culture with acetosyringone increased the regeneration and transformation efficiency by almost 10–12%. Followed by co-cultivation and washing, the infected calli were transferred to resting media for 5–7 days. This has been shown to increase the competence of call for better transformation and regeneration [39,40].

### 3.4. Optimization of Regeneration Medium and Measurement of Regeneration Frequency

**Figure 8:**
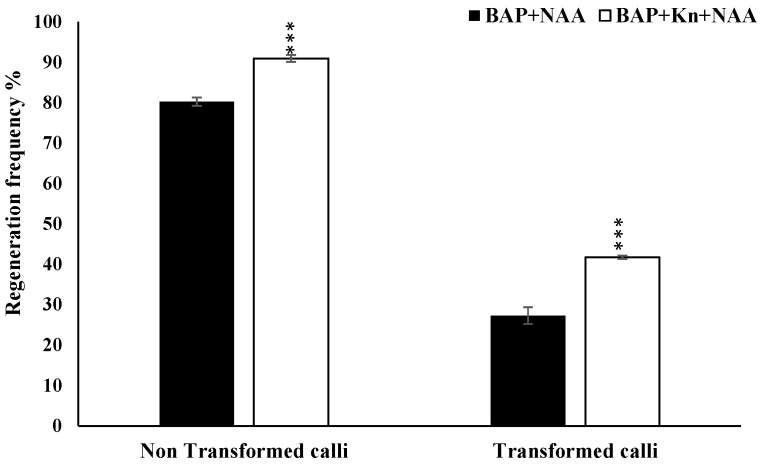
Effect of different hormones on regeneration frequency of non-transformed (regenerated without co-cultivation with Agrobacterium) and transformed calli (co-cultivate with Agrobacterium) of *indica* rice cultivar MTU1010. Asterisks (***) indicate statistically significant differences (*p* < 0.001).

**Figure 9:**
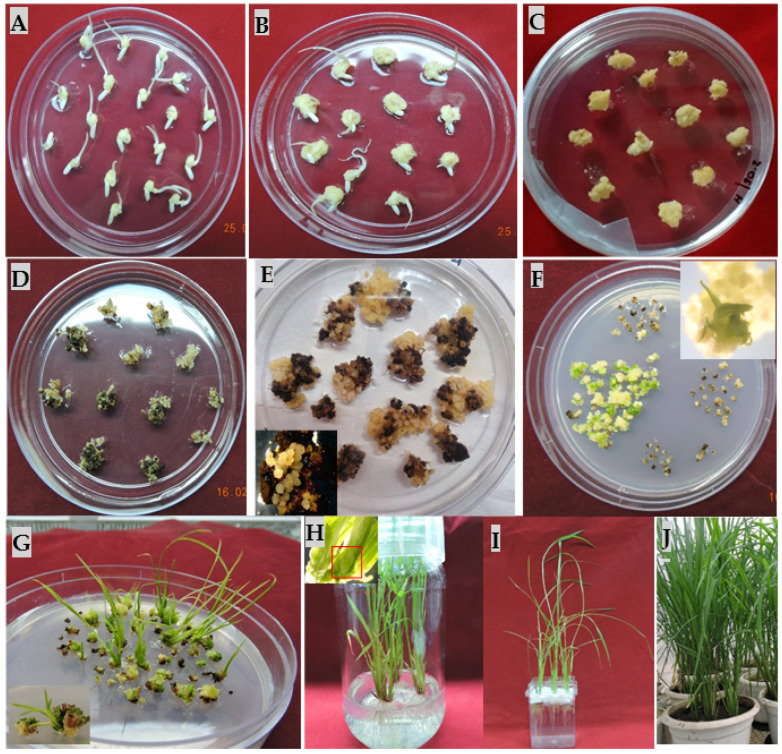
*Agrobacterium*-mediated transformation of MTU1010 rice with pCAMBIA1300-*P_AtRD29A_::OsIPT9* construct. (**A**) Mature seeds of MTU1010 in callus induction medium for 10 d; (**B**) 18 d old MTU1010 calli; (**C**) *Agrobacterium*-infected calli in CCM-2; (**D**) second selection; (**E**) third selection; (**F**,**G**) stereo microscopic view of emerging shoots from calli in regeneration medium; (**H**) rooting medium; (**I**) root hardening in Yoshida medium; (**J**) putative transgenic plants transplanted in soil and maintained at glass house facility.

To improve the regeneration efficiency of proliferated calli, various combinations of BAP, Kinetin, and NAA were tested in regeneration medium. It was observed that 2.5 mg/L 6-BAP, 1 mg/L kinetin, and 0.5 mg/L NAA was best-suited hormone combination for improved regeneration efficiency up to 91%, as compared to 80% in 2.5 mg/L BAP and 0.5 mg/L NAA in non-transformed calli of MTU1010. Further, this media composition was used for regenerating transformed calli. After the selection of calli for 40 days in hygromycin, the resistant calli were transferred on regeneration medium (RM-1) containing 35 mg/L hygromycin and 10 g/L Agarose. The agarose in regeneration media can reduce the water content [19]. A regeneration frequency of 42% was observed in the transformed calli cultured on MS medium and augmented with 2.5 mg/L BAP, 1 mg/L kinetin, and 0.5 mg/L NAA, while a 28% regeneration frequency was observed in media fortified with 2.5 mg/L BAP and 0.5 mg/L NAA (Figure 8; Supplementary Table S4). The emergences of green shoot buds were observed after 10–12 days of incubation (Figure 9). Most of the shoot buds were developed into shoots after 18–20 days. The proliferated shoots were transferred into rooting medium containing ½ MS salts and 20 g/L sucrose and 10 g/L glucose, which was found to be optimal for rooting in MTU1010. Sucrose provides a readily available carbon source for energy and growth during rooting.

A comparative study on different media compositions reported on *indica* cultivars has been summarized in Table 2.

Several factors of the culture media play a vital role in the tissue culture response of various rice genotypes, primarily *indica* cultivars, which are known to be recalcitrant to in vitro propagation and genetic transformation. For some genotypes, a particular concentration of growth regulators and nutrient source might stimulate proliferation while for others they might be unresponsive. Thus, in the present study, an attempt to nullify differential responsiveness to genotype variability has been attempted. A comparative analysis of previously reported media compositions was undertaken, exhibiting that different concentration of media were required for different rice cultivars. Barring the concentration of MS salts for CIM, an optimization has been made at individual successive propagation steps. Sucrose has been the primary choice of carbon source by independent groups [19]. However, in our study, maltose exhibited a comparatively higher embryogenic callus induction frequency. Auxin and cytokinin interactions are considered to be the most important regulation to induce organ development in the cultured tissues. For callus induction, 2,4-D in a range of 1.5–3 mg/L and BAP in rage of 0.10–0.25 mg/L has been used for optimization. Further, hormone variability was also assessed in regeneration medium in concentrations of BAP (1–4 mg/L), Kinetin (1–2 mg/L), and NAA (0.2–0.5 mg/L) [41,42,43,44]. For resuspension medium, pH and salt concentration were found to be governing factors for higher transformation efficiency. As well, pH 5.2 with various salts at a high concentration is routinely used [18,19]. However, we have found that, for ½ MS with 1.5% sucrose at pH 5.4, the lower ionic strength showed higher transformation efficiency, as evident from our results. Moreover, for rooting, only sucrose is used for most *indica* varieties [18,43,44], and our results exemplify that sucrose 20 mg/L and glucose 10 mg/L enhance rooting. Also, there is a major difference in the gelling strength of rooting media. A total of 4 g/L phytagel was commonly used in previous studies. However, in our study, the use of a lower gelling concentration of 2.5 g/L phytagel (lesser firm rooting media) has been found to be optimal for better root penetration and growth (Table 1). Furthermore, the supplementation of 0.05 mg/L NAA in rooting media promotes rooting.

### 3.5. Molecular Confirmation

**Figure 10:**
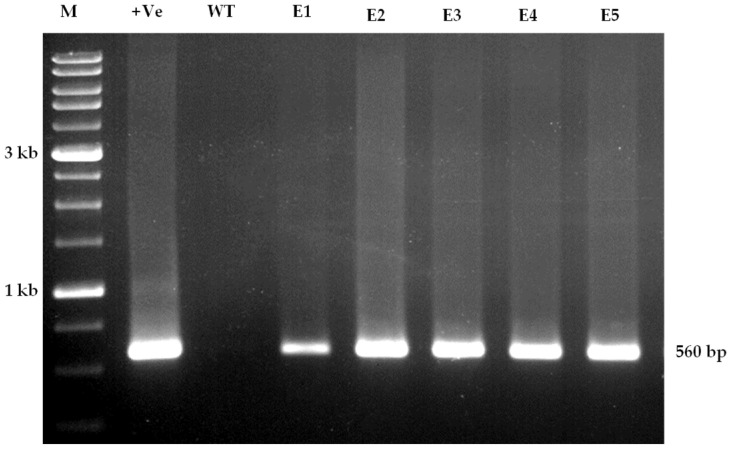
Molecular conformation of T_0_ transgenic plants using *hptII* gene-specific forward and reverse primers. Lane M: 1 Kb ladder; +ve: Positive plasmid; WT: Wild type MTU1010; E1–E5: Amplicon of *hptII* gene from corresponding putative transgenic events (Amplicon size—560 bp).

**Figure 11:**
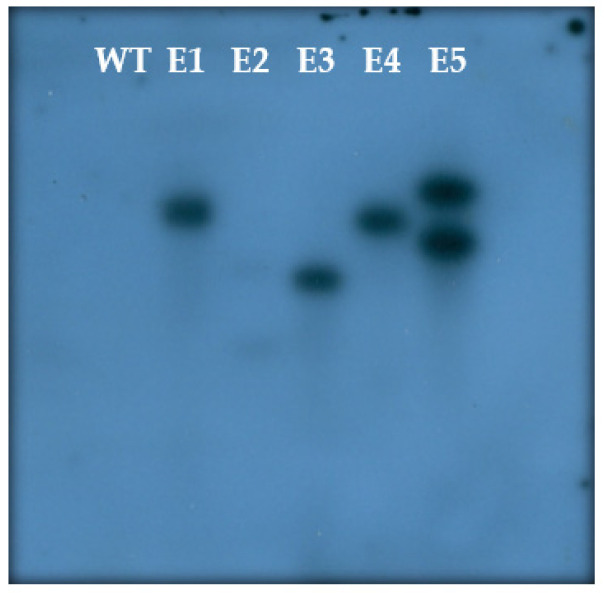
Southern blot analysis of T1 plants obtained from *Agrobacterium*-mediated transformation of MTU1010. Lane WT is the genomic DNA of wild type MTU1010 plant, Lanes E1–E5 are the five independent transgenic lines.

A transgene integration analysis was carried out on T0 plants and these were screened by PCR using hygromycin gene primers which showed an amplicon of 560 bp (Figure 10). DNAs from selected PCR positive progeny plants (T1) were digested with *Sac*I HF (NEB) enzyme to confirm for its selection marker gene (*hptII*) by Southern hybridization. We confirmed that the transgene inherited as a single copy insertion (#1, #3, and #4) and two copy insertions (#2 and #5) in *OsIPT9* transgenic lines (Figure 11). No hybridization signal was detected in the wild type MTU1010.

## 4. Conclusions

In the present study, successive steps of callusing, transformation, and propagation were optimized to attain maximum embryogenic calli, and a higher transformation efficiency and regeneration frequency, in rice mega variety MTU1010. The supplementation of cytokinin, BAP (0.25 mg/L), and with auxin 2,4-D (2.5 mg/L) resulted in higher embryogenic callusing with the highest response (92%) in CIM media containing maltose. Further, the infection and co-cultivation of *Agrobacterium* with a reduced salt concentration (RSM-2 and CCM-2) and pre-incubation of 30 min showed the maximum yield frequency of regeneration and transformation. The regeneration frequencies of transgenic MTU1010 plants were maximized on media with 2.5 mg/L BAP, 1 mg/L kinetin, and 0.5 mg/L NAA hormones. Following this protocol, we successfully developed *OsIPT9* stable transgenic rice plants which, in turn, were confirmed by *hptII* PCR and Southern hybridization. This protocol can prove instrumental in exploring the vast genetic diversity in rice, particularly recalcitrant *indica* genotypes, and uncover potential insights into gene annotation and crop improvement studies. This protocol and the approaches suggested will be useful for functional genomics and genome editing for the trait enhancement of *indica* rice cultivars.

## Figures and Tables

**Table 1:** Media used for *Agrobacterium*-mediated transformation of rice cv. MTU1010.

Name of the Media	Composition
Callus induction media (CIM)	MS Salts with B5 vitamins, 300 mg/L Casein hydrolysate, 560 mg/L L-Proline, 36 g/L Maltose monohydrate, pH-5.8, 4 g/L Phytagel. Autoclave the media and allow them to cool to room temperature and then add 2.5 mg/L 2,4-D and 0.25 mg/L 6-BAP
Yeast extract Mannitol medium (YEM)	Yeast extract 1 g/L, Mannitol 10 g/L, NaCl 1 g/L, MgSO_4_.7H_2_O 0.2 g/L, K_2_HPO_4_ 0.5 g/L, pH-6.8–7. Autoclave and store the media at room temperature
Yeast extract Mannitol Agar medium (YEMA)	To YEM medium, add 1.5% Agar
Resuspension medium (RSM-1)	MS salts with 1.5% sucrose and adjust the pH to 5.4 autoclave and store at room temperature
Resuspension medium (RSM-2)	½ MS or ¼ MS salts with 1.5% sucrose and adjust the pH to 5.4 autoclave and store at room temperature
Co-cultivation medium (CCM-1)	MS Salts with B5 vitamins, 300 mg/L Casein hydrolysate, 560 mg/L L-Proline, 36 g/L Maltose monohydrate, pH-5.8, 4 g/L Phytagel. Autoclave and then add 2.5 mg/L 2,4-D, 0.25 mg/L 6-BAP and 150 µM acetosyringone (freshly prepared)
Co-cultivation medium (CCM-2)	½ MS Salts with B5 vitamins, 300 mg/L Casein hydrolysate, 560 mg/L L-Proline, 36 g/L Maltose monohydrate, pH-5.8, 4 g/L Phytagel. Autoclave and then add 2.5 mg/L 2,4-D, 0.25 mg/L 6-BAP and 150 µM acetosyringone (freshly prepared)
Resting Media	To the CIM medium, add 300 mg/L Cefotaxime, 200 mg/L Timentin.
Selection medium-1 (SM-1)	To the CIM medium, add 300 mg/L Cefotaxime, 200 mg/L Timentin, 50 mg/L Hygromycin
Selection medium-2 (SM-2)	To the CIM medium, add 250 mg/L Cefotaxime, 50 mg/L Hygromycin
Regeneration medium (RM-1)	MS salts, 30 g/L Maltose monohydrate, pH-5.8, 1% Agarose. Autoclave the media and then add 2.5 mg/L 6-BAP, 1 mg/L Kinetin, 0.5 mg/L NAA, 250 mg/L cefotaxime and 35 mg/L Hygromycin
Regeneration medium (RM-2)	MS salts, 30 g/L Maltose monohydrate, pH-5.8, 0.8% Agarose. Autoclave the media and add 2.5 mg/L 6-BAP, 1 mg/L Kinetin and 0.5 mg/L NAA, 30 mg/L Hygromycin
Rooting medium	½ MS salts, 20 g/L Sucrose, 10 g/L Glucose, pH-5.8 then add 2.5 g/L Phytagel. Autoclave the media and then add 0.05 mg/L NAA
Root hardening	½ strength Yoshida Medium or ½ strength Hoagland medium
Acclimatization	Soil rite mixture
Transplanting	Soil

**Table 2:** Comparative study of different mediums used for agrobacterium mediated transformations in *indica* rice.

Media	Composition	[19]	[18]	[41]	[42]	[43]	[44]
Genotypes		IR64	CSR10, IR64, PB1, Swarna	ADT 43	AC39020	MTU1010	CO39
**CIM** **(Callus Induction Media)**	MS Salt C.H. L-Proline Maltose Sucrose Glucose Phytagel/Gelrite Agarose 2,4-D BAP pH	4.4 g/L 0.03 g/L 0.065 g/L --- 30 g/L --- 4 g/L 2 g/L 2.5 mg/L 0.15 mg/L 5.8	4.4 g/L 0.3 g/L 0.60 g/L 30 g/L --- 10 g/L 3 g/L --- 3 mg/L 0.25 mg/L 5.2	--- --- --- --- --- --- 3 g/L --- 2.5 mg/L --- 5.8	--- --- --- --- --- --- --- --- 2 mg/L --- 5.8	4.4 g/L 0.4 g/L 0.7 g/L 30 g/L --- --- 4 g/L --- 2.5 mg/L --- 5.8	4.3 g/L 0.3 g/L 2.8 g/L 30 g/L --- --- 3 g/L --- 3 mg/L --- 5.8
**Resuspension medium**	MS Salt Sucrose Glucose KCl MgCl_2_ Acetosyringone pH	4.4 g/L 68 g/L 36 g/L 3 g/L 4 g/L 150 µM 5.2	4.4 g/L 68 g/L 36 g/L 3 g/L 4 g/L 150 μM 5.2	Not Mentioned	Not Mentioned	Not Mentioned	4.3 g/L 68 g/L 36 g/L 3 g/L 4 g/L 150 µM 5.2
**CO-C** **(Co-Cultivation Media)**	MS Salt C.H. L-Proline Maltose Sucrose Glucose Phytagel Agarose 2,4-D BAP Acetosyringone pH	4.4 g/L 0.03 g/L 0.065 g/L --- 30 g/L --- 4 g/L 2 g/L 2.5 mg/L 0.15 mg/L 150 µM 5.8	4.4 g/L 0.3 g/L 0.60 g/L 30 g/L --- 10 g/L 3 g/L --- 3 mg/L 0.25 mg/L 150 µM 5.2	--- --- --- --- --- --- 3 g/L --- 2.5 mg/L --- 100 µM 5.8	Not Mentioned	4.4 g/L --- 0.5 g/L --- 20 g/L 10 g/L 4 g/L --- 1.5 mg/L --- 200 µM 5.2	4.3 g/L 0.3 g/L 0.60 g/L --- --- 10 g/L 4 g/L --- 3 mg/L --- 150 µM 5.8
**Selection Media**	MS Salt C.H. L-Proline Maltose Sucrose Glucose Sorbitol Phytagel Agarose 2,4-D BAP Hygromycin Cefotaxime Timentin Augmentin pH	4.4 g/L 0.03 g/L 0.065 g/L --- 30 g/L --- --- 4 g/L 2 g/L 2.5 mg/L 0.15 mg/L 50 mg/L 300 mg/L --- --- 5.8	4.4 g/L 0.3 g/L 0.60 g/L 30 g/L --- 10 g/L --- 3 g/L --- 3 mg/L 0.25 mg/L 50 mg/L 250 mg/L --- --- 5.2	--- --- --- --- --- --- --- 3 g/L --- 2.5 mg/L --- --- --- --- --- 5.8	Not Mentioned	4.4 g/L 0.4 g/L 0.7 g/L --- 30 g/L --- 1 g/L 4 g/L --- 3 mg/L --- 50 mg/L --- --- 600 mg/L 5.8	4.3 g/L 0.3 g/L 2.8 g/L --- 30 g/L --- --- 3 g/L --- 3 mg/L --- 50 mg/L --- 250 mg/L --- 5.8
**Regeneration Media (I)**	MS Salt Maltose Sucrose BAP NAA Kinetin Cefotaxime Augmentin Hygromycin Agarose pH	4.4 g/L --- 30 g/L 3 mg/L 0.5 mg/L 1 mg/L --- --- 40 mg/L 10 g/L 5.8	4.4 g/L 30 g/L --- --- 0.2 mg/L 2 mg/L 250 mg/L --- 30 mg/L 10 g/L 5.8	4.4 g/L 30 g/L --- 1 mg/L 1.5 mg/L 1.2 mg/L --- --- 30 mg/L 10 g/L 5.8	4.4 g/L 30 g/L --- 4 mg/L 0.5 mg/L --- --- --- 30 mg/L 10 g/L 5.8	4.4 g/L --- 30 g/L 2 mg/L 0.5 mg/L 1 mg/L --- 600 mg/L 30 mg/L 4 g/L 5.8	4.3 g/L 30 g/L --- --- 0.2 mg/L 2 mg/L --- --- --- 10 g/L 5.8
**Regeneration Media (II)**	MS Salt Maltose Sucrose BAP NAA Kinetin Cefotaxime Hygromycin Agarose pH	4.4 g/L --- 30 g/L 3 mg/L 0.5 mg/L 1 mg/L --- 40 mg/L 8 g/L 5.8	4.4 g/L 30 g/L --- 2.7 mg/L 0.5 mg/L 1.2 mg/L 250 mg/L 30 mg/L 8 g/L 5.8	Not Mentioned	Not Mentioned	Not Mentioned	4.3 g/L 30 g/L --- --- 0.2 mg/L 2 mg/L --- 30 mg/L 8 g/L 5.8
**Rooting Media**	MS Salt Sucrose Glucose NAA Cefotaxime Hygromycin Phytagel	4.4 g/L 20 g/L 10 g/L --- --- --- 4 g/L	2.2 g/L 30 g/L --- --- 250 mg/L 30 g/L 3 g/L	Not Mentioned	Not Mentioned	2.2 g/L 15 g/L --- --- --- --- 4 g/L	2.15 g/L 30 g/L --- --- --- 30 mg/L 3 g/L

## Supplementary Materials

The following supporting information can be downloaded at: https://www.mdpi.com/article/10.3390/mps6060113/s1, Table S1: Concentration of various plant growth regulators and antibiotics used; Table S2: Effect of supplement of hormones 2,4-D and 6-BAP on embryogenic calli induction in CIM media of MTU1010 mega rice variety; Table S3: Data represents the regeneration and transformation efficiency in different resuspension medium. Data shown are mean of three individual experiments; Table S4: Optimization of different hormones of the regeneration medium and its effects on regeneration frequency %.

## Abbreviation

CIM—Callus induction medium; O.D.—Optical density; 2-4D—2,4 dichloro phenoxy acetic acid; MS—Murashige and Skoogs medium; MS-B5—MS medium with B5 vitamins6-BAP—6 Benzyl amino purine; NAA—naphthalene acetic acid; C.H.—Casein hydrolysate; PGR—Plant growth regulator; RE—Regeneration efficiency; TE—Transformation efficiency.
