# Supplementary material for: A Versatile Protocol for Efficient Transformation and Regeneration in Mega Indica Rice Cultivar MTU1010: Optimization through Hormonal Variables
Source: Methods Protoc. 2023 Nov 23;6(6):113. doi: 10.3390/mps6060113 (PMC10745540; doi:10.3390/mps6060113)
Supplement: Supplementary file 1 [file mps-06-00113-s001.zip › mps-2549080-supplementary.pdf]

Table S1: Concentration of various plant growth regulators and antibiotics used.

**Growth Regulators/ Antibiotics**	**Abbreviations**	**Solvents**	**Stock concentration**	**Working concentration**
N^6-Benzyl amino purine	BAP	1N NaOH	1 mg/ml	2.5 mg/L
6-Furfuryl amino purine (Kinetin)	Kn	1N NaOH	1 mg/ml	1 mg/L
alpha- Naphthalene acetic acid	NAA	Absolute alcohol	1 mg/ml	0.5 mg/L
2,4-Dichlorophenoxyacetic acid	2,4-D	Absolute alcohol	---	2.5 mg/L
CEFOTAXIME SODIUM	Cefotaxime	Water	250 mg/ml	300 mg/L
TICARCILLIN DISODIUM / CLAVULANATE POTASSIUM	Timentin	Water	200 mg/ml	200 mg/L
3/5-Dimethoxy-4-Hydroxyacetophenone	Acetosyringone	DMSO	Freshly prepared	150 μM
Kanamycin	Kanamycin	Water	50 mg/ml	50 mg/l
Rifampicin	Rifampicin	Methanol	12.5 mg/ml	12.5 mg/l

Table S2: Effect of supplement of hormones 2,4-D and 6-BAP on embryogenic calli induction in CIM media of MTU1010 mega rice variety.

**Hormones Concentration**	**Replicates**	**No. of seeds inoculated**	**No. of seeds produced callus**	**Total no of embryogenic calli generated**	**Embryogenic callus induction (%)**
**CIM + (2.5 mg/L 2,4-D)**	**R1**	100	79	73	73
	**R2**	120	91	86	71.6
	**R3**	140	140	91	65.1
**CIM+ (2.5mg/L 2,4-D + 0.25mg/L BAP)**	**R1**	100	94	92	92
	**R2**	120	118	111	92.5
	**R3**	125	125	112	89.6

Embryogenic calli generation (%) = no. of embryogenic calli regenerated/no. of calli incubated × 100.

Table S3: Data represents the regeneration and transformation efficiency in different resuspension medium. Data shown are mean of three individual experiments.

Resuspension medium	Pre-incubated agrobacterium culture+acetosyringone	No. of calli used for transformation	No. of plantlet regenerated	Total number of Hyg^R plantlets	Regeneration frequency (a) (%)	Transformation efficiency (b) (%)
Full MS	0 min	250	21 ± 2.27	15 ± 1.69	7.9 ± 0.89	6.4 ± 0.67
	10 min	185	15 ± 0.72	14 ± 0.54	9.1 ± 0.38	7.5 ± 0.29
	30 min	200	35 ± 1.18	31 ± 1.18	17.5 ± 0.29	15.5 ± 0.59
1/2 MS	0 min	230	84 ± 1.78	74 ± 1.65	36.8 ± 0.77	32.5 ± 0.71
	10 min	200	86 ± 1.51	76 ± 1.44	43 ± 0.75	38.3 ± 0.72
	30 min	220	99 ± 2.27	91 ± 1.44	46.3 ± 0.96	41.9 ± 0.65
1/4 MS	0 min	250	91 ± 1.24	85 ± 1.88	36.9 ± 0.84	33.2 ± 0.85
	10 min	220	101 ± 0.98	92 ± 1.88	46.1 ± 0.81	40 ± 1.03
	30 min	200	101 ± 1.18	88 ± 0.78	50.7 ± 0.59	44.3 ± 0.30

(a) Regeneration frequency%= (Number of plantlets regenerated/Number of microcalli incubated) × 100

(b) Transformation efficiency%= (Number of hygromycin resistant plants/Number of calli co-cultivated with Agrobacterium) ×100

Table S4: Optimization of different hormones of the regeneration medium and its effects on regeneration frequency %.

Regeneration medium		Replicates	No. of calli used for transformation	No. of plantlet regenerated	Regeneration frequency (%)
3 mg/L BAP+ 0.5mg/L NAA	Non-transformed calli	R1	150	120	80
		R2	120	99	82.5
		R3	115	90	78.26
2.5 mg/L BAP+ 1mg/L Kinetin + 0.5 mg/ L NAA		R1	100	89	89
		R2	120	111	92.5
		R3	140	128	91.4
3 mg/L BAP+ 0.5mg/L NAA	Transformed calli	R1	110	33	30
		R2	108	24	22.2
		R3	145	43	29.6
2.5 mg/L BAP+ 1mg/L Kinetin + 0.5 mg/ L NAA		R1	125	52	41.6
		R2	100	41	41
		R3	150	64	42.6
